# Contextual influences on chronic illness: A multi-level analysis in the twin cities of Ramallah and Al Bireh in the occupied Palestinian Territory

**DOI:** 10.1016/j.healthplace.2021.102677

**Published:** 2021-11

**Authors:** Ahmad M. Alkhatib, Jonathan R. Olsen, Richard Mitchell

**Affiliations:** MRC/CSO Social and Public Health Sciences Unit, University of Glasgow, Berkeley Square, 99 Berkeley Street, Glasgow, G3 7HR, UK

**Keywords:** Urban context, Refugee camps, Green space, Health, Middle East, Multi-level methods

## Abstract

The features of the urban environment can support human health as well as harm it, but less is known about such influences in the context of middle eastern countries. The association between green space and the political classifications of the urban environment and the risk of chronic illness was investigated in a novel setting, the twin cities of Ramallah and Albireh in the occupied Palestinian territory. We used a generalised multi-level regression analysis to link the 2017 census data with Geographic Information System data. We modelled individuals at level one (n = 54693) and areas of residence at level two (n = 228), adjusting for individual demographic and socio-economic characteristics.

The proportions of ‘mixed’ trees in residential areas had a significant inverse association with the risk of chronic illness. On the political dimension, only living in a refugee camp had a significant positive association with chronic illness; however, this was largely explained and rendered non-significant when green space variables were entered into the models. Our ability to differentiate between several types of green space was important, as findings demonstrated that not all types were associated with reduced risk of chronic illness. Our results from a middle eastern setting add to the largely Western existing evidence, that trees in urban settings are important and beneficial to human health. Researchers and policymakers should pay more attention to the health consequences of refugee camps but also the role of trees in benefiting individuals' health in such a disadvantaged context.

## Highlights

• The study explored the urban political and environmental impacts on health in the Palestinian context.
• Living in a refugee camp is positively associated with the risk of chronic illness, over and above individual characteristics.
• Residential areas mixed trees are inversely associated with chronic illness, and explained the association with refugee camps.
• Not all types of green space are associated with a reduced risk of chronic illness.

## 1 Introduction

***“Health is created and lived by people within the settings of their everyday life; where they learn, work, play, and love.”*** - (The Ottawa Charter, 1986)

In every aspect of their lives, people are in constant interaction with their environment. Not solely physical interaction, but also emotional, social, political and cultural (Duncan and Kawachi, 2018). According to Krieger: “People literally embody, biologically, their lived experience, in societal and ecologic context, thereby creating population patterns of health and disease” (Krieger, 2011, page 125). Therefore, it is essential to investigate the influence of the environment to understand and improve population health (Frohlich et al., 2001). Today, more than half the world's population lives in urban environments, which is expected to reach 60% by 2030 (United Nations, 2018). The interactions between individuals and urban environments are complex. However, the potential for urban environments to support human health, as well as to harm it, is well recognised (Ompad et al., 2007).

Urban environments differ in the degree to which their influence on health has been researched. Whilst the influence of well-developed and slowly changing ‘western’ urban forms has been explored; there is substantially less research on new, rapidly growing, less developed urban areas. For instance, in the middle east and north Africa (MENA) region, urban environments are experiencing unprecedented environmental pressures and challenges, translated into environmental resource depletion, climate-related hazards, lifestyle modifications, congestion, and pollution (El-Zein et al., 2014). Yet, research assessing either positive or negative health impacts in these settings remains sparse, and which context-specific features are most related to health is not clear.

**Fig. 1:**
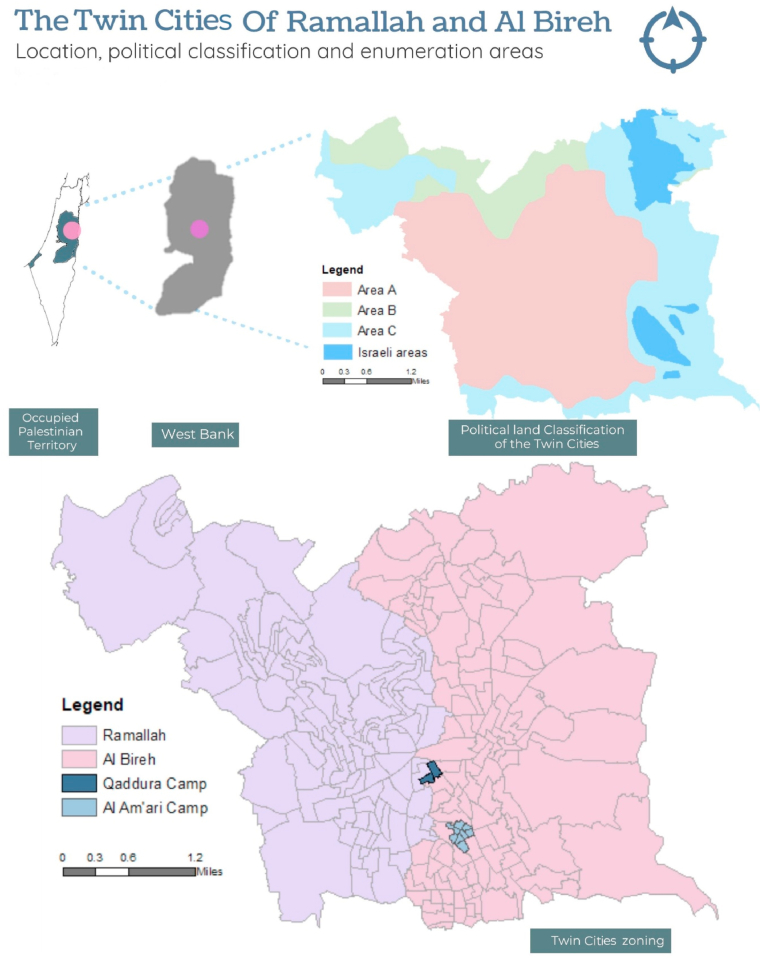
The twin cities of Ramallah and Al Bireh. (Sources: Administrative boundaries obtained from the Palestinian Central Bureau of Statistics in 2018, and the political classification (set in 1995) obtained from the ministry of local government in 2019).

This study addresses that gap by exploring the urban environment's impacts in a novel setting; the occupied Palestinian territories (oPt). The West Bank and the Gaza strip regions became known as the oPt after 1967 when Israel occupied them. The name oPt indicates that it is not an independent country. Located in the middle of the West Bank are the twin cities of Ramallah and Al Bireh (Fig. 1). Although physically a single settlement, each of the two localities has independent municipal services, headquarters and different socio-cultural structures. Ramallah can be considered less conservative and has more political and economic power than Al Bireh (Taraki, 2008). This study focused on the twin cities because they were ideal for assessing a relatively well-established environmental influence (urban green space) on health in an understudied setting and a potential influence specific to the area and context (political land classification and refugee camps). Thus, our study site and approach enabled us to investigate the urban environment and health relationships found within ‘conventional’ western urban areas for other parts of the world experiencing rapid urbanisation.

In general, the urban environment can influence health through three domains: the social environment, referring to the social structure, relations, and norms; the physical environment referring to features of the built environment such as buildings, green space, and physical infrastructure; and services or infrastructures such as health, social, municipal services as well as the local and governmental legislations and international agreements (Ompad et al., 2007). In the unique context of the oPt, this third domain extends to include the political environment related to the political conflict, the peace agreements, and the military occupation in the region that have an explicit health-related impact and roles in shaping all other domains.

### 1.1 Green space and health

Green space is an essential feature of the urban physical environment, and evidence has accumulated in the last decades of its various health benefits. Many studies found evidence that contact with green space is beneficial for a variety of health-related outcomes, for example, general health (Dadvand et al., 2016), longevity (Takano et al., 2002), life satisfaction (Olsen et al., 2019), mental health and well-being (Liu et al., 2020; McEachan et al., 2016; Zhang et al., 2020b), sleep (Astell-Burt and Feng, 2020a), obesity (Luo et al., 2020), birthweight (Torres Toda et al., 2020), and mortality (Rojas-Rueda et al., 2019). Neighbourhood green space is also associated with reduced prevalence of different chronic diseases and morbidities, such as cardiometabolic and cardiovascular conditions (Brown et al., 2016; Dalton and Jones, 2020), type 2 diabetes mellitus, hypertension, stroke and asthma(Dalton et al., 2016; Maas et al., 2009; Mitchell and Popham, 2008; Twohig-Bennett and Jones, 2018).

There are several intertwined pathways via which green space might influence health and well-being (Hartig et al., 2014): it directly affects health by providing salutogenic resources and restoring psychological and physical health. For example, green space reduces rumination (Lopes et al., 2020), promotes immunity (Rook, 2013) and may also reduce harmful exposures, such as air pollutants, heat and noise (Rook, 2013; Wolch et al., 2014). It also offers an *indirect* influence by promoting and facilitating healthy lifestyles through impacts on intermediate factors such as behaviour, capacities and decisions; for example, green space increases the opportunity for physical activity and social interaction (Hartig et al., 2014; Kondo et al., 2018; Markevych et al., 2017). Although some of these effects have been proven via experimental designs (Markevych et al., 2017; Ward Thompson et al., 2012), there remains substantial variation in the evidence base for both presence and magnitude of effects in the real world.

Studies of the relationship between green space and health are mostly limited to cities in highly developed countries in the global North (Kondo et al., 2018; Markevych et al., 2017; Shuvo et al., 2020; Zhang et al., 2020a). Given that these associations may be highly affected by the broader geographical, social, economic, cultural, and built environments of these settings, the lack of evidence from less-developed, rapidly urbanising areas of the developing world is problematic (Chiabai et al., 2020; Rojas-Rueda et al., 2019; Shuvo et al., 2020; van den Berg et al., 2015). For example, studies revealed that associations between health and green space are likely to be affected by the socio-cultural context, as it plays a vital role in how people perceive green space, interact in it, and embody its effects (Kearns and Milligan, 2019; Markevych et al., 2017). Other studies implied context-specific characteristics moderate the link between green space and both individual and population health. Such as the degree of urbanity (Mitchell and Popham, 2007), dependency on cars, and green space abundance (Richardson et al., 2010), It is likely that green space would also hold importance for health in urban areas of the developing world. One of the aims of this study was to explore the relationship between green space and health outcomes in a middle eastern context. A second aim was to consider the influence of social and political context.

### 1.2 Refugees, refugee camps and political land classification

In the 1948 war, several hundreds of thousands of Palestinians (the number is disputed) were uprooted from their land and became 'refugees' (Shalhoub-Kevorkian, 2015). A proportion of these refugees were concentrated in ‘refugee camps' in the West Bank and the Gaza strip. As with the rest of the West Bank, the twin cities of Ramallah and Al Bireh hosted refugees within refugee camps (Fig. 1). In the years/decades following, more affluent refugees left the camps and settled in the Palestinian cities, whereas the less advantaged continue to live in these refugee camps to this day, in a structurally disadvantaged context (Aruri, 2015; Helu, 2012; Taraki, 2008). It is important to note that both refugees inside and outside the camps are still formally labelled as having a refugee background regardless of where they now reside.

After the Israeli occupation in 1967, the Palestinians were under total Israeli military control, experiencing oppression, discrimination, and injustices, resulting in the first uprising (“Intifada” in Arabic) in 1987. It ended in 1996, following the Oslo II interim peace agreement in September 1995 between Palestinian Liberation Organization (PLO) and Israel. This agreement granted the Palestinians an ambiguous form of sovereignty over large cities in the West Bank, dividing land into three political area types: Area A (covering 17.7% of the West Bank area), in which the Palestinian Authority has total administrative and security control; Area B (18.3%) under Palestinian civil administration but remaining under Israeli security control; and Area C (61.1%) over which Israel has full administration and security control (B'tselem, 2019; Jad and Hilal, 2011; Khamaisi, 2018). As part of the West Bank, the twin cities of Ramallah and Al Bireh were subjected to this political land classification and divided into these three areas (Fig. 1). After the Oslo accords between 1993 and 1995, the twin cities became the governmental, economic, and cultural centres of the West Bank. This led to some economic and political stability, which accelerated demographic and economic growth and increased immigration. However, improvements were concentrated in the areas classified politically as A or B. These zones are administered by the Palestinian Authority and are more autonomous than area C, controlled by the Israeli administration. Area C also has more significant restrictions on individuals and development and proximity to conflict points such as Israeli settlements and checkpoints. To a lesser extent, Areas A and B have better institutional resources and infrastructure than areas C (Helu, 2012). Such unusual cultural, economic, planning, and political differences could plausibly influence health over and above individual characteristics (Macintyre et al., 2002). Many aspects of life in the oPt, specifically the twin cities, are affected by this political environment via military occupation, geographic fragmentation, and political segregation. It also affects the distribution of green space. For instance, refugee camps are crowded “informal” politically created settlements that lack green space (Helu, 2012). In contrast, restrictions on development in the areas politically classified as C have resulted in relatively *more* green space. Open green space is often a target for development in areas classified as A and B, resulting in increased pressures on green areas (Helu, 2012).

### 1.3 Aims and objectives

This study was designed to explore the following:

a. How much variability in the probability of chronic illness is attributed to the areas of residence? And how much of this variability is explained by the natural and political area characteristics?
b. Is the risk of chronic illness associated with living in the context of a refugee camp, Al Bireh or Ramallah and the political land classifications?
c. Is the risk of chronic illness associated with the proportion of green space within areas of residence?

## 2 Methodology

The study design linked census data for individuals and households, green space data, and political boundaries with the probability of chronic illness using a multi-level methodology.

### 2.1 Study area: the twin cities of Ramallah and Al Bireh

#### 2.1.1 Geography and population in the twin cities in 2017

The twin cities of Ramallah and Al Bireh have an area of 40.7 $km^{2}$ and are divided into 228 enumeration areas (small areal geographical boundaries defined by the Palestinian Central Bureau of Statistics - PCBS) (Fig. 1). The PCBS created enumeration areas for census data collection, and in this study, they were used as a proxy for ‘neighbourhood’. The average size of an enumeration area was 0.17 $km^{2}$ and the mean number of people residing in each one was 359. Ramallah has 105 enumeration areas and a total population of 34,786. Al Bireh has 113 enumeration areas and a total population of 41,574.

#### 2.1.2 Refugee camps in 2017

The twin cities include two refugee camps (Fig. 1) established in 1948–1949. Al Amari refugee camp in Al Bireh consists of 8 enumeration areas and has 4690 residents. Qaddura refugee camp in Ramallah consists of just two enumeration areas and has a total population of 850. These two refugee camps are characterised by crowding, poor infrastructure, and poor housing quality (Helu, 2012). Alamari refugee camp is under the jurisdiction of the United Nations Relief and Works Agency, while Qaddura refugee camp is governed by a services committee (ARIJ, 2012a; 2012b).

### 2.2 Data

This study used individual, household, and area-level variables. The individual and household variables were obtained from the PCBS 2017 census data. The census included the entire population of the twin cities, including the refugee camps (n = 86527), conducted in August 2017 through an interview with every household's head (main financial supporter). The response rate was 94.6% (n = 81900). It included demographic, socio-economic, and health variables as well as household characteristics.

Several census variables used in this study were restricted to individuals aged 14 and above. Therefore, individuals below this age were excluded from the analysis (n = 25034). Individuals who had zero years of residence (n = 997) were also excluded because they represented individuals not living in the twin cities (living in Israel or abroad or new arrivals). Missingness was 1%, and a complete case analysis was, therefore, suitable (Tabachnick and Fidell, 2014). The final analytical sample was 54693 individuals living in 228 enumeration areas.

A two-level structure was established in which data about individuals and households were treated as level one and data about enumeration areas as level two. The median number of individuals per enumeration area was 238, ranging from 56 to 470. Households were not treated as a separate level because doing so requires methods out of this study's scope (Josephy et al., 2016; Raudenbush, 2008).

Area-level variables (described below) were obtained from several sources. The enumeration area boundaries were obtained from the PCBS, green space obtained (and edited) from the Applied Research Institute Jerusalem (ARIJ), the political boundaries from the ministry of local government. Area-level data were linked with the individual-level data by enumeration area codes.

### 2.3 Measures and descriptions

#### 2.3.1 Outcome variable

The main outcome variable was self-reported medically diagnosed chronic illness requiring ongoing treatment. This was measured in the census as a binary ‘yes/no’ variable. Participants were asked: “do you suffer from any medically diagnosed chronic illness and receive a continuous treatment” with possible answers of yes or no. The number of individuals who said yes was 5039 (9.2%).

#### 2.3.2 2Area-level variables

**Green spaces:** Green space data obtained from ARIJ were collected in 2011 and contained some incomplete sections. They were updated for this study using an up-to-date base map (2018) with a resolution of 1 m per pixel, obtained from the ministry of local government (2019) to match the dates when the census data were collected. The update was performed using geographic information system software ArcGIS v.10 and the methodology provided in supplementary material 1.

**Fig. 2:**
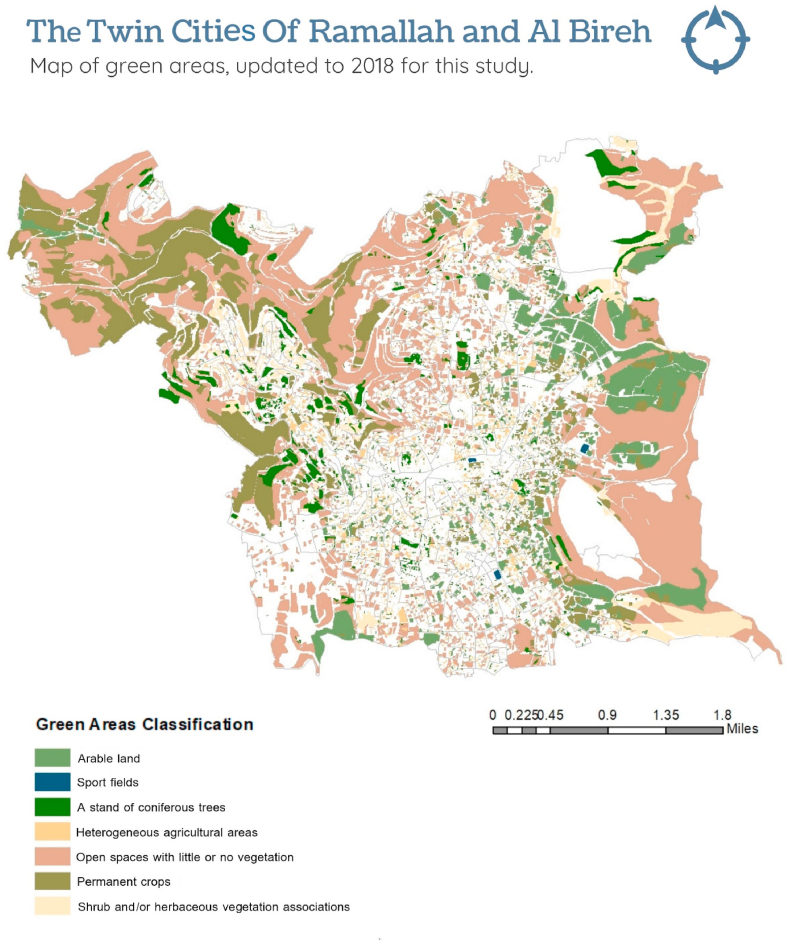
Map of green areas in the twin cities, updated to 2018 for this study.

The green spaces map delineated seven green space categories: arable land, used/suitable for agriculture; open space with little or no vegetation and which is not used/ideal for agriculture; sports grass fields; permanent crop trees including agricultural trees (primarily olive trees); stands of trees, including different types of coniferous trees; heterogeneous trees, including various types of agricultural and non-agricultural trees; shrublands and/or herbaceous vegetation, including different types of small-sized shrubs and/or herbs (Fig. 2). The proportion of land area covered by each category of green space in every enumeration area was calculated by intersecting the enumeration area boundaries (Fig. 1) with the green space map (Fig. 2). This process identified that some categories were sparse; therefore, similar green space types were combined to create three new categories: Open space (containing open space with little or no vegetation, arable land, sports fields, and shrub and/or herbaceous areas), permanent crop trees, and mixed trees (containing coniferous and heterogenous trees). Crop trees were not combined with mixed trees because of the difference in structure and accessibility compared to the mixed trees category. Crop trees are relatively small trees and are found mainly in less accessible areas.

The three green space variables were modelled as continuous, indicating the proportion of land in each enumeration area they covered. A description of these variables is presented in Table 1.

**Table 1:** Median and interquartile range of green space categories in the total of 228 enumeration areas.

Category	Median	Interquartile range
Open space	20.1%	20
Crop trees	0.15%	5.3
Mixed trees	6.8%	8.5

**Localities:** This variable described the area context in terms of the administrative or municipal management body and the composition of the three areas of the twin cities: Ramallah, Al Bireh and the refugee camps. The locality variable with three categories: Ramallah, Al Bireh, and refugee camps, as seen in Fig. 1. Numbers and percentages of individuals in each category are presented in Table 2. Both the locality and the political classification (below) were obtained from the ministry of local government.

**Table 2:** Description of locality and political classification.

Item	Population count	%
Locality		
Ramallah	23317	43%
Al Bireh	27701	50%
Refugee camps	3675	7%
Political class		
A & B	47577	87%
C	7116	13%

**Political classification:** Each enumeration area was defined as either area A, B or C based on the dominant proportion of the political classification (Fig. 1). Since only 11 enumeration areas were classified as type B, area type B was combined with area A. Both are governed by the Palestinian authorities, in contrast to area C, controlled by Israeli authorities. The political classification was a binary variable where one indicated area C and 0 indicated area A or B (Table 2).

#### 2.3.3 Individual-level variables

Demographic and socio-economic characteristics were included in the models to control for their effect on chronic illness risk. These variables were obtained from the PCBS 2017 census. Continuous variables were age with a mean of 36.1 years (sd:16.3; range:14–98), years of residence in the current place of residence with a mean of 24.8 years (sd:17.2; range:1–98), and a household assets scale with a mean of 0.04 (sd:0.78; range: 1.73-1.68). There is no standard measure of household affluence or income in the oPt, so a household assets scale was created for this study, using a common methodology to reflect household socio-economic conditions (Poirier et al., 2020). The scale included thirteen variables from the 2017 census describing the presence or absence of household resources: TV, microwave, freezer, vacuum cleaner, drying machine, dishwashing machine, water filter, home library, internet connection, tablet/iPad, laptop/pc, central heating and air conditioning. These variables were chosen based on their socio-economic relevance, use in similar studies, and household variability. Scale creation used principal component analysis (PCA), rather than simply summing the number of assets (which had a mean of 6.4 and range 0–13) because PCA uses appropriate weighting for each asset (Kolenikov and Angeles, 2009). Since each item was a binary measure, conventional linear PCA was not appropriate. Instead, PCA based on a tetrachoric correlation matrix was conducted (Basto and Pereira, 2012). The Cronbach alpha of the tetrachoric correlation matrix was 91%, indicating good internal consistency (Gadermann et al., 2012).

The description of the categorical variables at the individual and household levels is presented in Table 3. In some cases, small numbers in categories necessitated categories to be merged for the analysis. The participation in labour force variable included separate categories for ‘*employed’* and ‘*unemployed’* as those in the labour market, and separate categories for ‘*housework’*, ‘*students’*, and ‘*other’* as those out of the labour market but with likely different lifestyles. Both original and combined categories are reported in Table 3.

**Table 3:** Individual and household characteristics of the total sample and by the presence of chronic illness.

Item	Original coding	Merged coding	Count	%	Chronic illness N/%
Sex					
	Female	Female	27581	50.4%	2478(9.1%)
	Male	Male	27112	49.6%	2561(9.3%)
Refugee status					
	Registered refugee Unregistered refugee	Refugee	27365	50%	3031(11%)
	Not refugee	Not refugee	27328	50%	2008(7.3%)
Health insurance					
	Governmental, UNRWA, private, combination, Israeli, other	Yes	44531	81.4%	4443(10%)
	No	No	10162	18.6%	596(5.8%)
Participation in the labour force					
**In the labour force**					
	Categories of work hours	Employed	26141	47.8%	1725(6.6%)
	Unemployed	Unemployed	1980	3.6%	100(5%)
**Out the labour force**					
	Studying	Studying	11293	20.6%	130(1.1%)
	Housework	Housework	10315	18.9%	1119(10.8%)
	Old or disabled Income without work Retired Other	Other	4964	9.1%	1965(39.6%)
Educational attainment					
	Diploma BA Master PhD	University	23658	43.3%	1538(6.5%)
	11–12 years (high school)	High school	11314	20.7%	757(6.7%)
	7–10 years (preparatory)	preparatory	12321	22.5%	960(7.8)
	1–6 years (primary)	Primary	4665	8.5%	771(16.5%)
	Illiterate Literate (no education)	No education	2735	5%	1013(37%)
Marital status					
	Married	Married	31876	58.3%	3680(11.5%)
	Single Engaged	Single	20344	37.2%	526(2.6%)
	Divorced Separated Widowed	Other	2409	4.5%	833(33.7%)
Residential type					
	Apartment Room Caravan/shack	Apartment	46357	84.8%	3924(8.5%)
	House Villa	House or Villa	8336	15.2%	1115(0.13%)
Tenure					
	Own	Own	40627	74.3%	3732(9.2%)
	Rent furnished Rent non-furnished Exchange of work	Rent	14066	25.7%	1307(9.3%)
Private car ownership					
	Count	One or more cars	35757	65.4%	2581(7.2%)
		No car	18936	34.6%	2458(13%)

#### 2.3.4 Multi-level analysis

A multi-level modelling approach was used as the tool best suited to account for both inter-individual and inter-cluster variation (Austin and Merlo, 2017; Diez-Roux, 2000; Garson, 2014; Leyland and Groenewegen, 2020). Generalised linear multi-level (hierarchical) models were conducted in R v4.0.2 (R Core Team, 2020), using the *lme4* package (Bates et al., 2015). The fitted models' estimates were obtained based on a maximum likelihood estimation technique using Laplace approximation (Finch et al., 2015; Li et al., 2011; Raudenbush et al., 2000). The clustering of individuals in the enumeration areas was treated as a random effect, accounting for cluster dependency (Snijders and Bosker, 2012).

The following models were performed to assess the association between the probability of chronic illness and the area-level characteristics while accounting for confounders at the individual level:

**Model 1**: the **null** model, showing the variance in chronic illness between enumeration areas without accounting for the characteristics at the two levels.
**Model 2**: including only the area-level political characteristics, without adjustment for individual-level confounders.
**Model 3**: including the area-level political and green space characteristics, without adjustment for individual-level confounders.
**Model 4**: the **baseline** model including individual-level characteristics, without the area-level characteristics, showing the importance of the control variables.
**Model 5**: including the area-level political characteristics while adjusting for individual-level confounders.
**Model 6**: the **final** model adjusted for all characteristics at the two levels. When compared to model 3, it assessed the addition of area-level characteristics after adjusting for confounders at the individual level.

All the explanatory variables were centred around the grand mean to enhance computing and reduce multicollinearity (Enders and Tofighi, 2007). Population average odds ratios and confidence intervals are approximated from the multi-level models and reported in the results instead of the cluster-specific estimates because they have a more straightforward and intuitive interpretation (Austin and Merlo, 2017). The population average odds ratios are interpreted as comparing two individuals from two different enumeration areas but sharing all other characteristics except the variable of interest.

The generalised linear regression with logit link fixes the variance of the individual level-1 at $\pi^{2}/3$ or 3.29; therefore, the variance at the enumeration area level is not comparable between models with different individual-level components (Austin and Merlo, 2017). To allow for appropriate comparison between models, in the models which included individual-level covariates, the variance at the enumeration area level was rescaled based on the variance of the linear predictor, a method proposed by Snijders and Bosker (2012). Two types of pseudo R squared were reported to show the variance explained by the models: the marginal $R_{\mathrm{dicho}}^{2}$ or the adjusted total variance explained by the covariates**,** and the conditional $R_{\mathrm{dicho}}^{2}$ or total variance explained by both the covariates and the clustering of individuals in enumeration areas (Jaeger et al., 2017; Nakagawa and Schielzeth, 2013). Additionally, two measures are presented to assess the clustering effects: the interclass correlation coefficient (ICC) and the median odds ratio (MOR). The ICC estimates the residual contextual effect or the proportion of variation in the probability of chronic illness attributed to the enumeration areas. The MOR assesses the magnitude of the contextual effects on an odds ratio scale (Austin and Merlo, 2017). Finally, the model fit statistics are reported by the deviance.

## 3 Regression results

Table 4 reports the six models’ outputs with the enumeration area-level variables. The null model showed that without accounting for any characteristics, 13.4% of the variance in the propensity for chronic illness was attributed to the clustering effect of the enumeration area-level. The MOR for the enumeration area-level indicates that if an individual moved from an enumeration area with a lower risk of chronic illness to an enumeration area with a higher risk, their risk of ‘in median’ could increase 1.98 times. This is a useful way to quantify the effect of the enumeration area on the odds of chronic disease. Model 2 added area-level political characteristics to the null model. These variables explained 1.2% of the total variance and 7.2% of the variance at the enumeration area-level in the propensity of chronic disease. There was evidence of an association between chronic illness and residence in refugee camps. Adding these characteristics reduced the variance attributed to enumeration area-level to 12.4%, and the MOR decreased to 1.93.

**Table 4:** Population average odds ratios and 95% confidence intervals from multi-level models for the analysis of the association between enumeration area characteristics and the risk of chronic illness.

Model OR^a^ (95% CI^b^)	1 Null	2	3	4 Base-line^c^	5	6 Final^c^
**Area characteristics**						
Ramallah		*reference*	*reference*		*reference*	*reference*
Al Bireh		1.19 (0.99–1.44)	1.12 (0.93–1.35)		1.13 (0.91–1.40)	1.03 (0.84–1.27)
Refugee camps		**2.14 (1.41**–3.27) **	1.47 (0.94–2.32)		**1.91 (1.17**–3.09) **	1.38 (0.83–2.29)
Political class A&B		*reference*	*reference*		*reference*	*reference*
Political class C		0.84 (0.64–1.11)	0.87 (0.65–1.16)		0.89 (0.65–1.21)	0.77 (0.56–1.06)
% of Mixed trees			**0.97 (0.96**–0.99) **			**0.96 (0.95**–0.97) **
% of crop trees			1.01 (0.99–1.02)			1.01 (0.99–1.02)
% of open space			**0.99 (0.98**–1.00) **			0.99 (0.99–1.01)
**Enumeration Area effect**						
Variance	0.51	0.473	0.423	0.359	0.344	0.293
ICC	13.4%	12.4%	11.4%	9.4%	9.0%	7.7%
MOR	1.98	1.93	1.86	1.77	1.75	1.68
**Model statistics**$R_{\mathrm{dicho}}^{2}$						
*Marginal*	/	1.2%	2.5%	38.3%	38.9%	40.2%
*Conditional*	13.4%	13.6%	13.6%	47.7%	48%	48%
Deviance	2484	32470	32448	23604	23596	23564

** P-value<0.01; *P-value<0.05.

a OR-population average odds ratio.

b CI-confidence interval (upper-lower).

c Controlling for: age, sex, education, participation in labour force, marital status, refugee status, health insurance, years of residence, household car ownership, and household assets index. The log-odds estimates for these variables are shown in the supplementary material 2.

Model 3 added the green space variables to model 2, explaining 2.5% of the total variance and 17.1% of the variance at the enumeration area-level in chronic illness propensity. The association between refugee camps and chronic illness risk was considerably attenuated and no longer statistically significant after including green space variables. A higher proportion of mixed trees and open space in the areas of residence were significantly associated with lower levels of chronic illness, without accounting for the individual-level variables.

In models 4, 5 and 6, individual-level characteristics were included. The variables that did not improve the model fit (tested via a likelihood ratio test and the deviance) were discarded; residential type and tenure. In baseline model 4, the individual-level variables were added to the null model, explaining 38.3% of the total variance and 29.6% of the variation in chronic illness propensity between enumeration areas. Most of these variables were associated with the probability of chronic illness. They were important confounders for the relationship between the enumeration area-level characteristics and the likelihood of chronic disease. The estimates of the individual level are shown in supplementary material 2.

In model 5, the area-level political variables were added while adjusting for the individual-level characteristics, explaining 39% of the total variance in the probability of chronic illness and 32% of the variance at the enumeration areas level. Compared to model 2, the association between residence in refugee camps and chronic illness was attenuated but persisted (OR 1.91 CI [1.17–3.09]). There was no difference in odds of chronic illness for those residents in Al Bireh, compared with Ramallah, or for residence in political classifications C, relative to A and B (combined).

The final model included variables at both levels, explaining 40.2% of the total variance and 42.5% of the variance at the enumeration area-level in the probability of chronic disease. In addition to the clustering at the enumeration areas, all the explanatory variables explained 48% of the variance overall. The proportion of mixed trees in an enumeration area was associated with chronic illness, with a 4% reduction in odds for a 1% increase in the proportion of mixed trees (OR 0.96 CI [0.95–0.97]). To understand this association's size, consider the range of proportions of mixed trees in the enumeration areas; 0.1%–40.7%. The final model suggests that living in an enumeration area in the 1st quartile range (around 2.8%) is associated with two times higher probability of chronic illness than living in enumeration areas in the 3rd quartile (about 9%), all else being equal.

None of the political variables or the other two green space categories (open space and crop trees) was associated with chronic illness risk. The association between living in refugee camps and chronic illness was again attenuated and non-significant when adding green space variables to model 5. Whilst being careful to note these are associations rather than 'effects', we conclude that only the proportion of mixed trees in the areas of residence has a significant association with chronic illness. In comparison to the baseline model (only with control variables), the final model shows that adding the area-level characteristics explained 18.4% of the variability at the area level. Accounting for the individual and the area-level characteristics in the final model, the remaining variance in chronic illness attributable to the between enumeration areas variation was 7.7%. In odds ratio terms, the enumeration areas with a higher risk of chronic illness have a median, 1.68 times higher odds compared to enumeration areas with lower risk, all else being equal.

## 4 Discussion

This study aimed to explore urban green space and political features in the twin cities of Ramallah and Al Bireh, oPt, in relation to population health, measured by the likelihood of chronic illness. The analysis linked environmental variables to census data via small area geographical boundaries. Models assessed the association between risk of chronic illness and area characteristics, accounting for the individuals' dependency on their areas of residence and adjusting for several important confounders.

Just over 13% of the variation in the probability of chronic illness was attributed to the enumeration area of residence. Contextual characteristics were found to explain 18.4% of the variation in the likelihood of chronic illness at the enumeration area-level while accounting for the individual and household characteristics which, themselves, explained 29.6% of this variation. We observed the proportion of land cover with ‘mixed trees’ as inversely associated with chronic disease risk. Residing in a refugee camp was associated with an increased risk of chronic disease; however, this association was sharply attenuated and non-significant when green space characteristics were included. Finally, no evidence was found for an association between political land classification and chronic health. Within the confines of a cross-sectional, observational study, the findings shed light on the possible role of the urban environment context as a determinant of health, irrespective of individual-level factors.

### 4.1 Greenspace

Our study builds upon previous literature on green space exposure and health. A recent systematic review found evidence of an inverse association between surrounding green space in a home buffer zone and all-cause mortality (Rojas-Rueda et al., 2019). Evidence is also present for chronic health conditions (Brown et al., 2016; Dalton and Jones, 2020; Maas et al., 2009; Massa et al., 2016; Twohig-Bennett and Jones, 2018). Not all studies found evidence of inverse associations between green space and chronic illness. For example, a longitudinal study conducted in Doetinchem in Netherland found no association between self-reported chronic illness with the percentage of green space in a 1 km radius around the residence place (Picavet et al., 2016). Lack of evidence is most likely due to methodological issues rather than the absence of association. However, in the urban, global north and west, the expectation would generally be for a positive relationship, whereas, studies stemming from developing countries that have the highest burden of diseases, the evidence is weak, mainly due to the low number and low quality of studies (Shuvo et al., 2020).

Amid the uneven distribution of evidence, our study was important because it addressed an understudied and different social, cultural, and climatic context. This was the first study investigating the association between green space and chronic illness in a middle eastern country. According to two recent systematic reviews for the Lower Middle-Income Countries literature, only one study has sought association between green space and another health-related outcome in this region (Rigolon et al., 2018; Shuvo et al., 2020). This study was conducted in Egypt and found no association between green space and obesity (Mowafi et al., 2012). Given that obesity is considered an important risk factor for chronic illness and similarities between the current study and Mowafi's in terms of the cultural context, multi-level methodology and confounders included, this was a surprising result. However, Mowafi et al. had a much smaller sample (3546 individuals in 50 areas) and did not differentiate between trees and other types of green spaces. Our study only found an association with one of the three green space measures we examined. Indeed, since most of the previous work on the relationship between health and green space has not separated specific types of greenery, our ability to do so was also important.

Our results support the limited existing research highlighting trees as a particularly important form of urban nature (Astell-Burt and Feng, 2020b; Donovan et al., 2013; Kardan et al., 2015; Reid et al., 2017). For example, while cardiometabolic diseases constitute the largest share of chronic illness burden in the West Bank (Mosleh, 2018), cross-sectional and longitudinal studies found that tree canopy is negatively associated with cardiometabolic conditions (Astell-Burt and Feng, 2020b; Kardan et al., 2015). While Reid et al., 2017, found that trees have a positive association with self-reported health compared to grasslands that do not have an association. Mitchell's study of associations between mental health and outdoor physical activity also found woodlands and trees to be particularly important (Mitchell, 2013).

Two possible explanations could support the importance of trees. First, non-tree vegetation tends to be affected by seasonal fluctuations, especially in arid climates such as that in the middle eastern region, where the lack of rain and water resources only permit such areas to be green around the spring season. In contrast, trees' resistance and conditioning in such a climate present them as a consistent source of greenery. Second, trees have added benefits in terms of their effect on possible pathways between health and green space, such as reducing urban noise and pollution, providing shade, and more visible greenery than non-tree vegetation (Kardan et al., 2015). On the other hand, this study found that not *all* tree classifications were protective of health. Crop agricultural trees (primarily olive trees) are abundant in the twin cities. However, they are mainly located in the peripheries or hills of the twin cities or their less accessible isolated areas. Further, crop trees are not diverse. These issues of accessibility and diversity have been highlighted as potentially important factors in determining a beneficial influence of green space on public health (Rook, 2013; van Dillen et al., 2012; Wheeler et al., 2015). We recommend that future studies explore the accessibility and diversity of green space in more depth and explore intermediate factors such as air pollution and noise.

### 4.2 Political context

The analysis revealed that living in a refugee camp increases the odds of chronic illness by 91%, compared to Ramallah residents, adjusting for individual characteristics. Previous studies have shown that living in refugee camps increases chronic illness odds (Jonassen et al., 2018). Scholars have elaborated on the challenges posed by residence in refugee camps (Helu, 2012; Miari, 2012; Taraki, 2008), including inadequate infrastructure, poor housing conditions, extreme overcrowding and adverse socio-economic situation. Our study suggests that the urban environment's physical features, as captured by green space, have a role in explaining the relationship between living in a refugee camp and health.

The political land classification was hypothesised to be associated with the risk of chronic disease; however, the results did not support this hypothesis. This was unexpected as such divisions potentially impact multiple aspects of life such as stability, development, exposure to political violence and many others (Fields, 2010; Isaac et al., 2015; Khamaisi, 2017; Parsons and Salter, 2008). There is no directly comparable literature against which to set our findings. It could be argued that the effects of the political land classifications as a source of power territoriality and fragmentation on health was not well-captured in the context of the twin cities of Ramallah and Al Bireh, a centralised urban setting. For example, access to health care may not have been affected dramatically by political classification in this setting, given the proximity in different locations of the twin city. However, further data are required to confirm this assumption. Perhaps the influence of political classification would be better captured in peripheral sites, such as remote villages and rural areas in the West Bank, or even in other localities, where this designation impacts more aspects of life. Further research is needed, with a broader geographical scope.

## 5 Strengths and limitations

A key strength of this study was its novel setting. Furthermore, including the political area characteristics as a unique constituent of the oPt allowed us to compare the literature from other settings to assess the importance or the unimportance of local-specific features. Few studies have examined the influence of neighbourhood characteristics, including green space, in the Middle Eastern region, and this is the first in oPt. Hence it begins to fill a gap in the literature about such contexts. It used a comprehensive measure of the quantity and type of green space, obtaining a high-resolution green space map for small areas that provided a geocoded quantitative description of *all* the green space in the enumeration areas. As a result, different green spaces types were quantified, including open lands with natural vegetation, parks, public gardens, private/domestic gardens, and street trees. This distinction between different types of green space answers the multiple calls for such an approach in the literature (Markevych et al., 2017; Zupancic et al., 2015). The analysis was based on the census data with a high response rate, and covered all the population in the twin cities. The study's multi-level methodology accounted for the dependency between individuals and different spheres of the environment, by which individuals share common characteristics with others in the same cluster. Accounting for the hierarchical structure of the environment and various individual factors reduced the possible effects of potential confounders, biases and the indirect selection that could negatively impact the results.

However, as with many studies using area-level measures, the study is subject to the “uncertain geographic context problem” because it relied on administrative boundaries that were not designed for research purposes (Kwan, 2012). The assumption was that the areal units of residence adequately captured individuals ‘exposure to their environment’. The study was also subject to all cross-sectional studies' limitations, including the temporality in the relationship between green space and health and potential overestimation of the effects due to direct selection, by which individuals favouring area greenness may choose to live in areas with high proportions of green space. The quality of green space was not evaluated, which may impact the relationship between green space and health. Another limitation was being based on a self-reported measure for chronic illness and not specifying diagnosed disease. No information was available on different types of conditions. The study may have missed those suffering from undiagnosed/unmedicated chronic diseases. Finally, data on other important aspects of the urban environment such as air pollution and traffic were not available. Future work should try to include these since they are related to, and interact with, green space.

## 6 Conclusion

This study contributes to the literature on the relationship between the urban environment and health within the developing countries by providing evidence from a different and understudied climatic, political, and cultural setting. We found evidence of associations between contextual characteristics and the risk of chronic illness in the twin cities of Ramallah and Albireh in the oPt. This study highlighted that the greenspace type is important. Areas with a higher proportion of trees were associated with lower levels of chronic disease, irrespective of individual-level factors. These findings highlight the importance of differentiating between types of green space to better decipher and assess the potential protective influence of green space and population health, mainly how this might vary within rapidly developing contexts.

Finally, this study highlights the broader importance of the political determinants of health, showing the adversity associated with living in politically created contexts such as refugee camps on chronic disease outcomes. Researchers and policymakers should give more attention to green space in disadvantaged contexts such as refugee camps to reduce health inequalities. Future studies should adopt a longitudinal methodology to implement evidence of causality.

## Declaration of competing interest

The authors declare no conflict of interest.
