# Supplementary material for: Contextual influences on chronic illness: A multi-level analysis in the twin cities of Ramallah and Al Bireh in the occupied Palestinian Territory
Source: Health Place. 2021 Nov;72:102677. doi: 10.1016/j.healthplace.2021.102677 (PMC8633762; doi:10.1016/j.healthplace.2021.102677)
Supplement: Multimedia component 1 [file mmc1.docx]

|  |  |  |
| --- | --- | --- |

## Green space data obtained from ARIJ and the editing methodology

The green space data was obtained from ARIJ in November 2019 and it is for the land cover in the twin city of Ramallah and Al Bireh in the year 2011/2012. The data obtained from ARIJ is set on the coordination system projection of Israel TM Grid, which was changed to Palestine 1923 grid, to be compatible with other data from other different sources.

ARIJ data includes three overall categories: agricultural areas, artificial surfaces and forests and semi-natural areas. Within these categories the data are split into 7 subcategories as the following:

- Arable land: areas suitable for agriculture even if it is not all cultivated.
- Permanent crops: agricultural trees, mainly olive trees
- Heterogeneous agricultural areas: a mix of different kinds of agricultural vegetation
- Artificial surfaces: sports grass fields
- Forests: refers mainly to concentrations of coniferous trees such as Pinus and Cupressus trees
- Shrub and/or herbaceous vegetation: small or medium size bushes that may be associated with other wild vegetations mainly ‘Pittosporum spinosum’ with other wild woody herbs such as Thymus and Salvia species.
- Open spaces with no or little vegetation: not used open space and poorly suitable for agricultural uses.

The map obtained from ARIJ including the seven green space sub-classifications is shown in Figure 4. Figure 4 also shows Israeli areas (in red) which are restricted to Palestinians (surrounded by a separation wall), these areas include green and built-up areas but the green areas that exist inside Israeli areas are not included in the map.


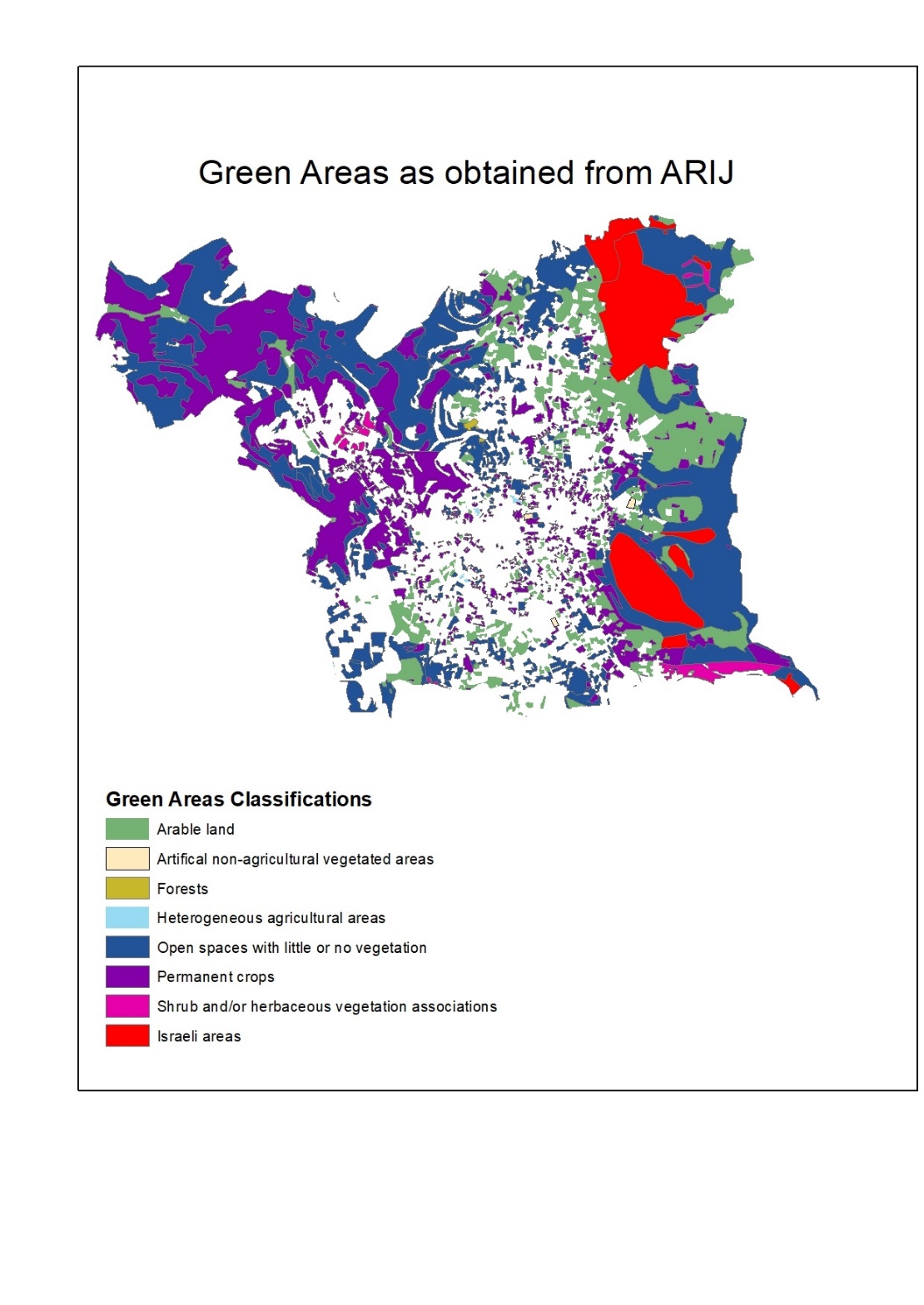


Figure 4:Green areas map obtained from ARIJ

After exploring the quality of data and comparing it with aerial photographs from Environmental Research Institute’s (ESRI) ArcGIS, Google maps and Google street view, it appeared that the land cover data obtained from ARIJ needs editing and modifications. Because green space data needs to be as much accurate and complete as possible, so that it does not affect the results of this research, two reasons backed the decision to edit the data obtained from ARIJ: First, because the data from ARIJ was collected in 2011-2012, whereby the census data used in this study is for the year 2017, and a considerable amount of change in the built-up areas took place in 7 years in the fast-growing twin city of Ramallah and Al Bireh . Second, the data itself had some problems in terms of completeness and in some instances incorrect classification was present.

## Editing Methodology

Editing was performed in Environmental Systems Research Institute’s (ESRI) ArcMap software v. 10.3, in the projected coordination system of Palestine 1923 grid. Before updating the green space data, a data layer for buildings and roads, obtained from the municipalities, was used to remove the new built-up sections that took place in the 7 years between the old and the new data from the land cover data obtained from ARIJ. This procedure was performed by overlaying the built area map layer and green space layer and then erasing the buildings and roads from the original land cover map. This created a new land cover map with updated built-up areas. The next step in updating the green area layer in the twin city by manual editing, in a systematic methodology.

To compare the green space map from ARIJ with the green space on the ground, a high-resolution orthogonal (aerial) photograph of the region of the twin city of Ramallah and Al Bireh obtained from the ministry of local government and dated to June 2018, was used. The high-resolution of 1 meter per pixel image facilitated editing at a fine scale, permitting mapping small patches of green space, such as street trees and around houses and buildings in addition to differentiating between green space categories. This image is the primary source of information in the editing process, however, in the instances where there was an uncertainty on the type of green space, Google street view was used as the definitive source of information.

To ensure a systematic editing approach, a fishnet editing grid was used with a cell size of 1000 meter square over the whole area of the twin city. Using the grid columns starting from the east toward the west, each cell was edited from the north toward the south, to prevent missing any cell in the grid.

In each cell, the green space data layer obtained from ARIJ was compared to the orthogonal satellite imagery, and whenever there are differences such as a new built-up area or a new street, updating was made. Editing was also made in some instances where the ARIJ data was incomplete or improperly labelling green space categories. How the different categories of land cover were verified is explained below.

### Arable land, open space with little or no vegetation and shrub areas and/or herbaceous vegetation associations

The three categories that do not include trees are arable land, open space with little or no vegetation and shrub areas. Arable land does not mean that the land is vegetated with agricultural plants, in most instances were not vegetated and these were identified by the uniform brown or any markers of agricultural activity such as markers of ploughing, whereby vegetated arable lands were identified by the uniform striped, green colour as markers of planted vegetables or other harvests. The open spaces with little or no vegetation were identified by the presence of white or grey spots on the land indicated rocky or chalky soil, whereby areas that include paved or unpaved roads, car parks, construction sites, and dumpster sites that contain solid or construction debris wastes were not added to the open space category. It’s worth mentioning here that both arable land and open space categories may contain a few single scattered trees or shrubs in a large open space.

For the shrub areas and/or herbaceous vegetation associations, these were identified from scattered dark green colour, mainly in a circular shape. Below are example images form the orthogonal image and google street view of the categories of arable land (Figure 5), open space with little or no vegetation (Figure 6) and shrub areas and/or herbaceous vegetation associations (Figure 7).

| 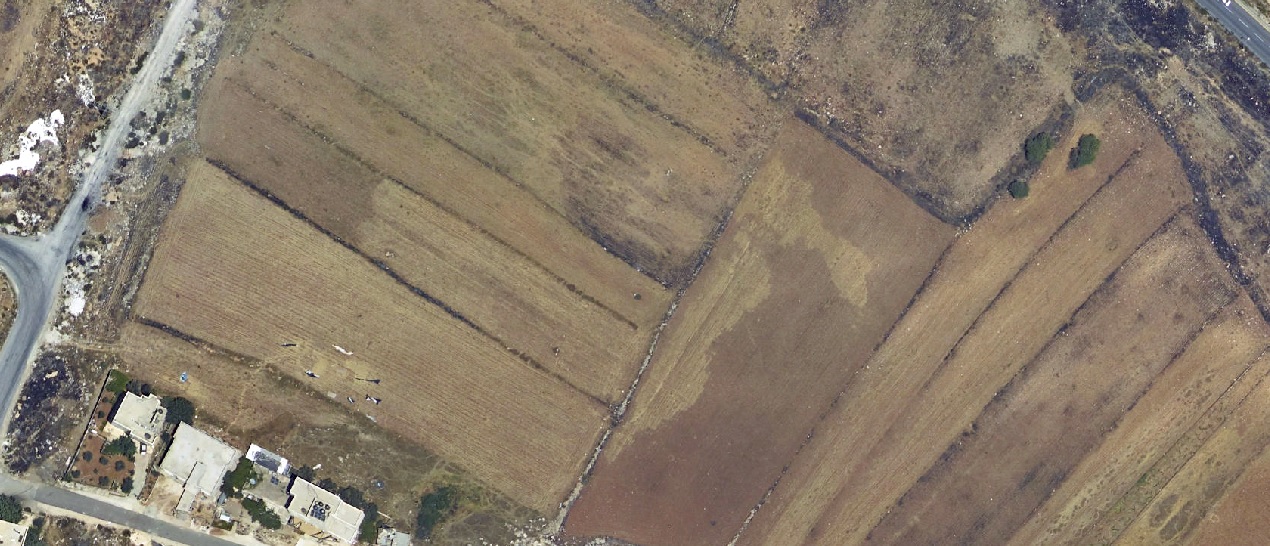 |
| --- |
| 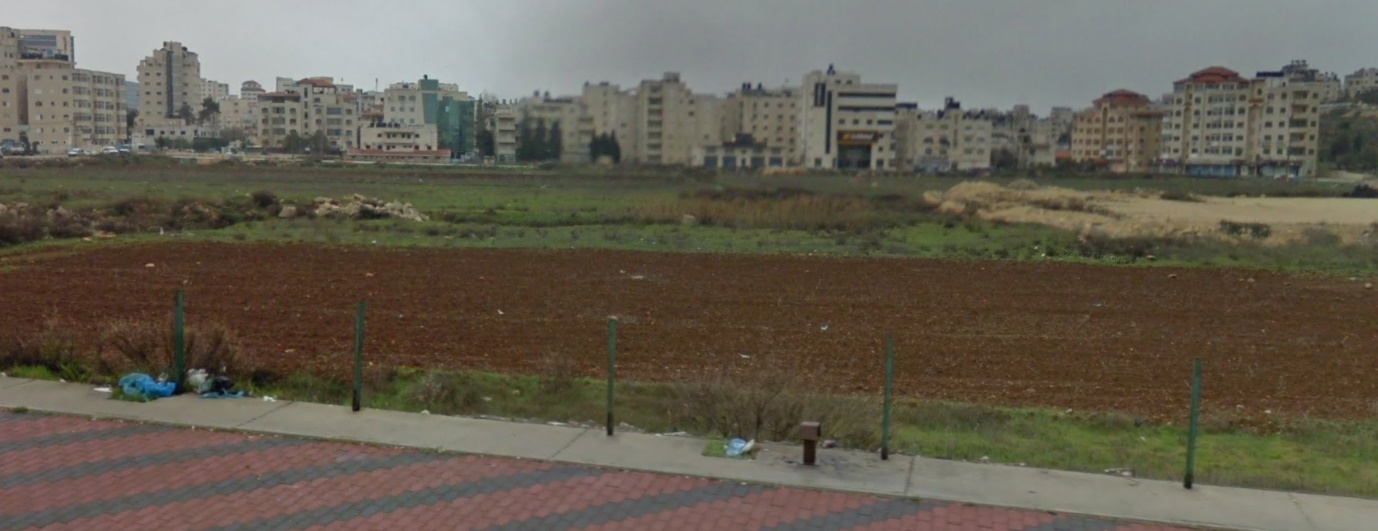 |
| Figure 5: Arable Land in the orthogonal and Google street view image |
| 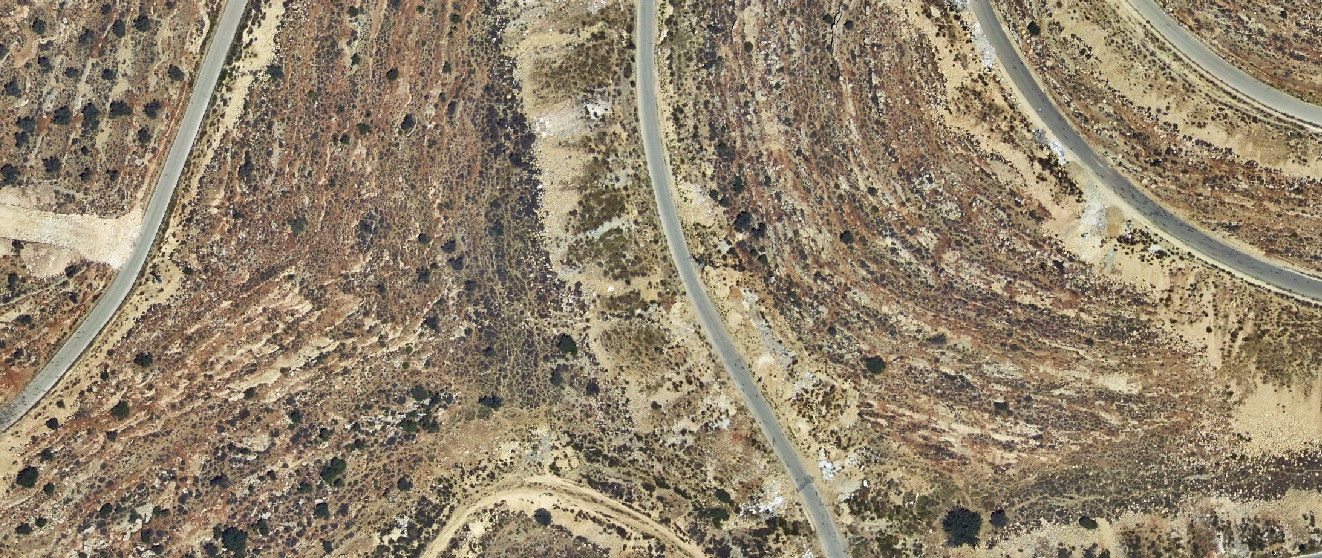 |
| 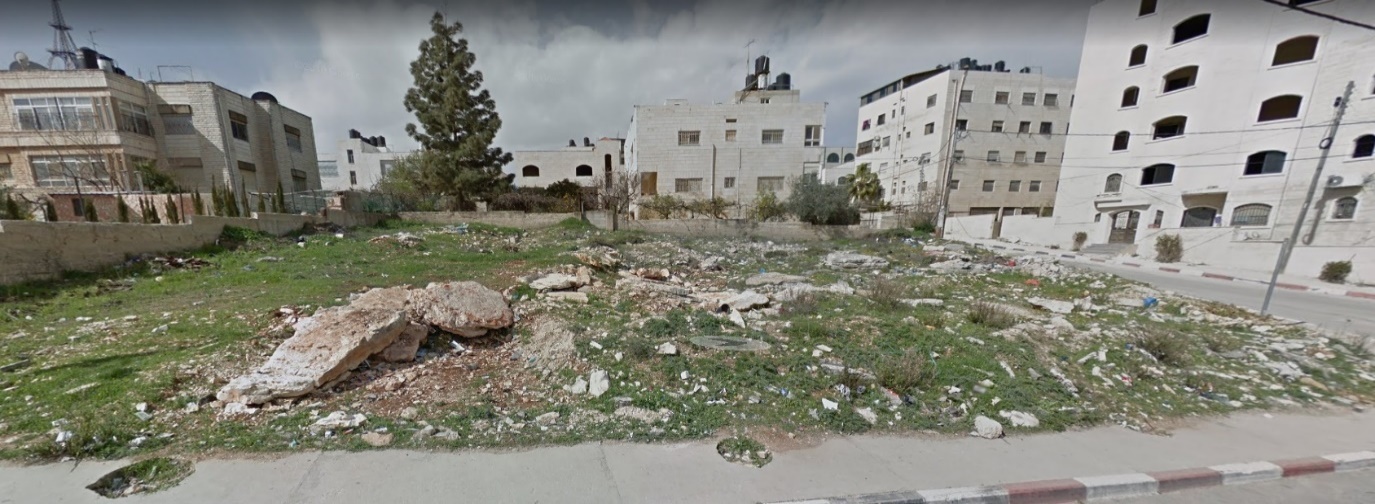 |
| Figure 6: Open space with little or no vegetation in the orthogonal and Google street view image |
| 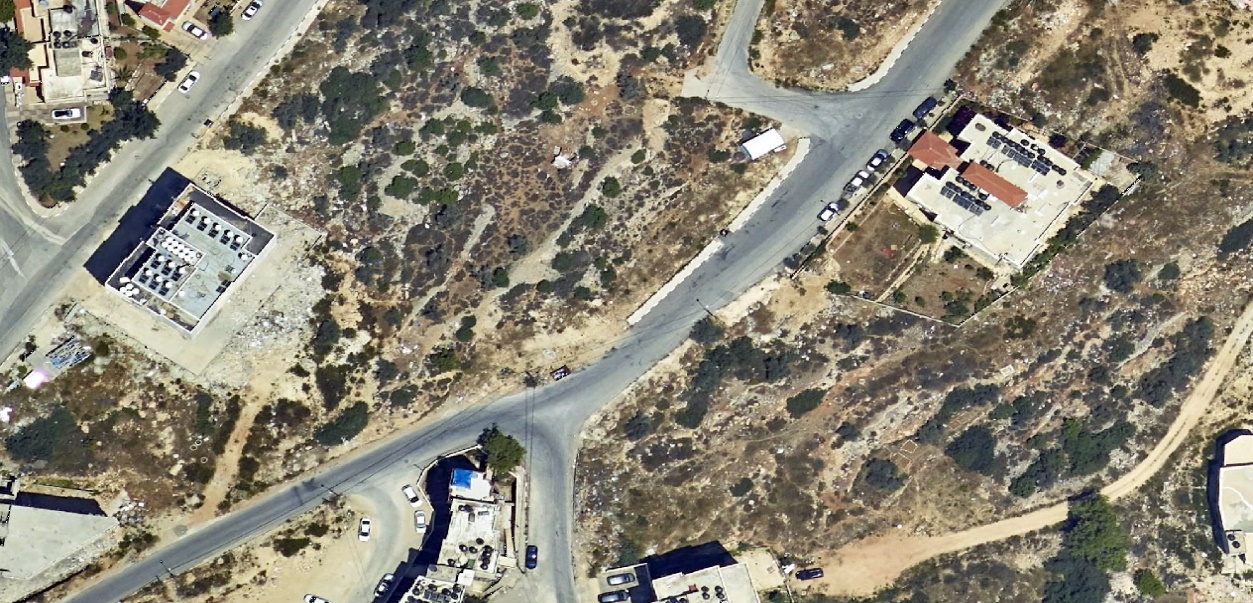 |
| 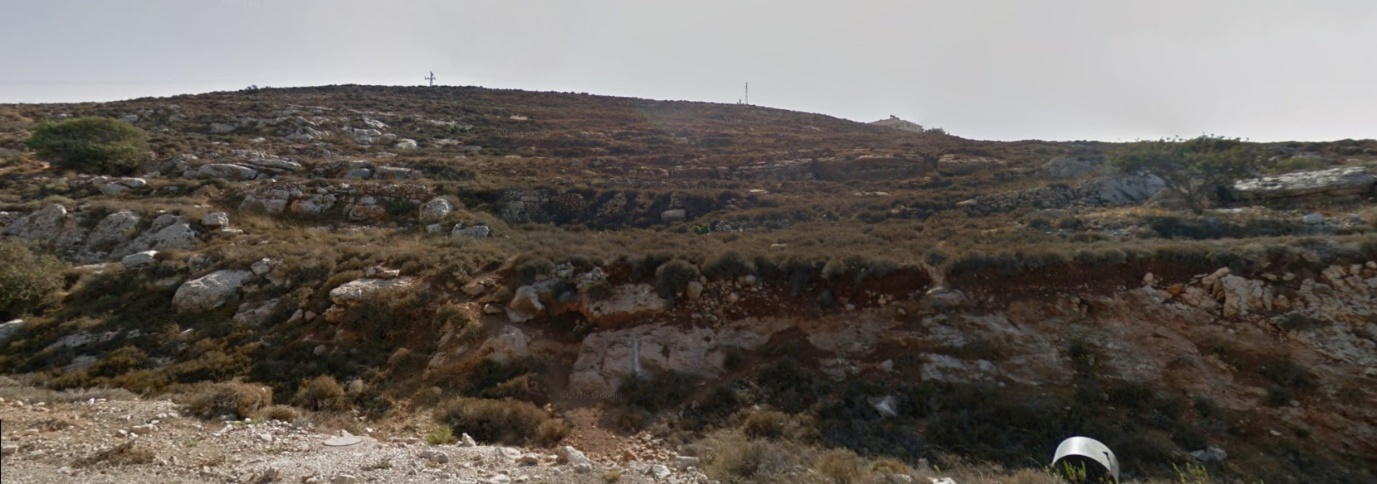 |
| Figure 7: Shrub areas and / or herbaceous vegetation associations with little or no vegetation in the orthogonal and Google street view image |

### Forests, permanent crops and heterogenous agricultural areas

Forests, permanent crops and heterogenous agricultural areas categories are the categories that include trees. In the data obtained from ARIJ, there is a category of land cover named forests, which refers to areas with cuneiforms trees. However, actual forests do not exist in the twin city of Ramallah and Al Bireh and because the name of the category is misleading, as commonly forests are defined as referring to large areas or a dense mass of trees, it was changed to a stand of trees to reflect small patches of coniferous trees.

The three categories of trees differ in terms of the type of trees that they consist of. As mentioned earlier a stand of trees consists of coniferous trees, permanent crop areas are predominantly olive trees, (but on a very few occasions it may consist of almond and fruit trees), and heterogeneous agricultural areas include a mix of different types of trees and may be associated with other kinds of vegetation such as grasses, flower beds and ornamental trees. Heterogenous agricultural areas mainly present beside or around residential structures, such as houses and buildings. This category is nearly exclusively a privet or domestic vegetation, unlike a stand of trees or permanent crops categories which are open tree plantation.

While editing, the difference between these categories was based on the pattern, sizes, and colour of trees. The permanent crops are recognized mainly based on the uniform pattern of the planted trees in addition to the colour of the soil, which is normally brown, resembling an arable land. Unlike a stand of trees which is less patterned, and the soil colour is greyer caused by the presence of rocks and wild herbs. In addition to that, the sizes of trees in a stand of trees are larger compared to permanent crop trees. While different tree sizes and different tree colours imply that it is a heterogeneous “agricultural” area, including a mix of trees from all types. Below are the images of the stand of trees (Figure 8), crop trees (Figure 9), and heterogeneous trees (Figure 10) from the orthogonal image and Google street view.

| 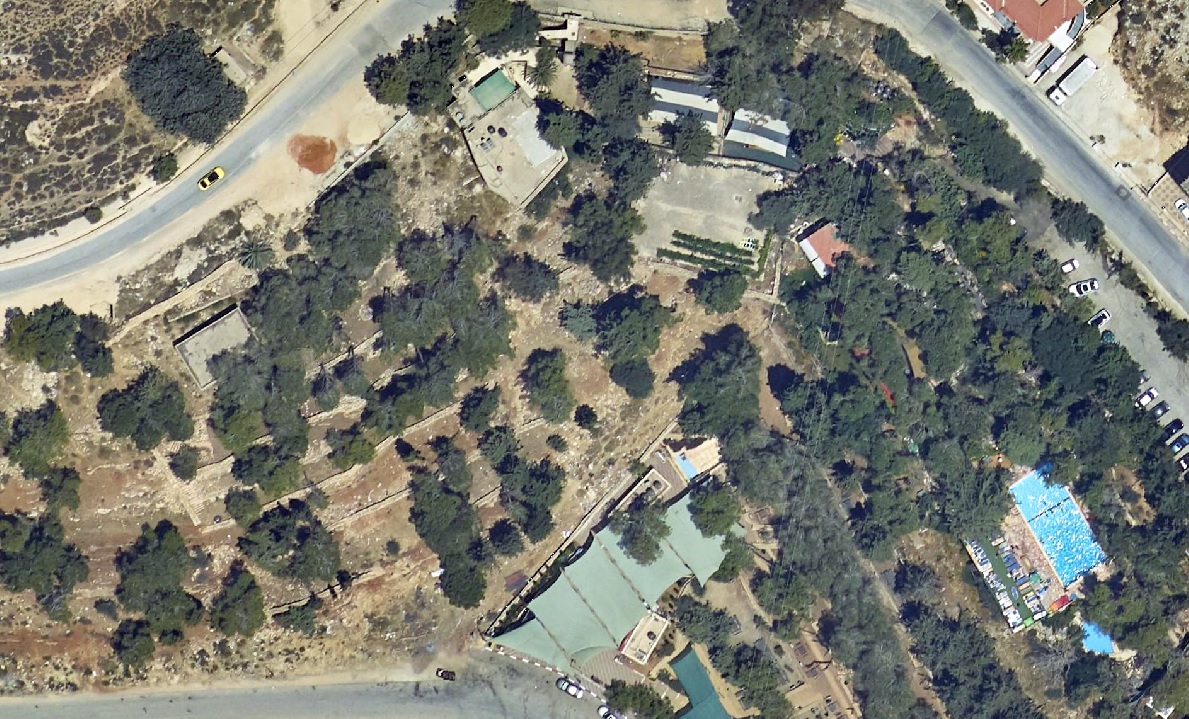 |
| --- |
| 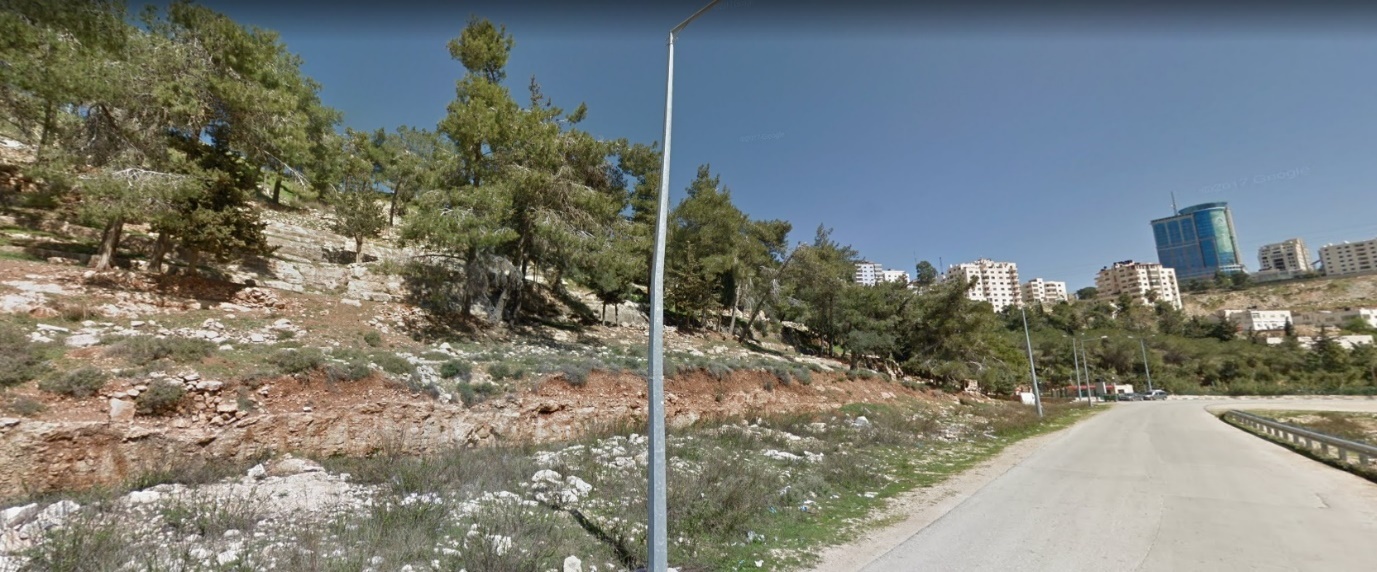 |
| Figure 8: A stand of trees as appears in the orthogonal and Google street view image |
| 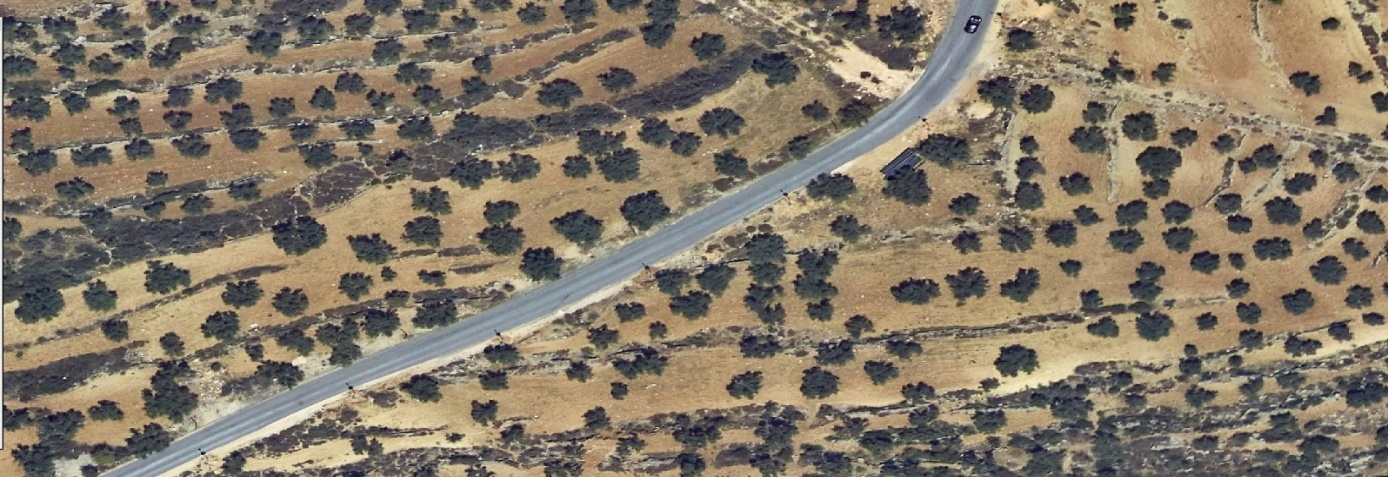 |
| 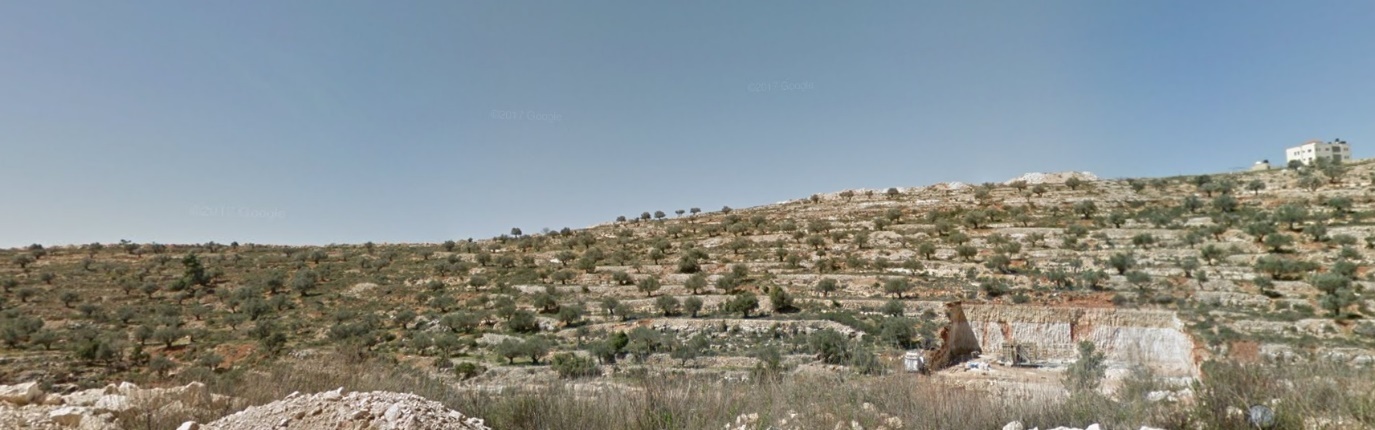 |
| Figure 9: Permanent crops as appears in the orthogonal and Google street view image |

| 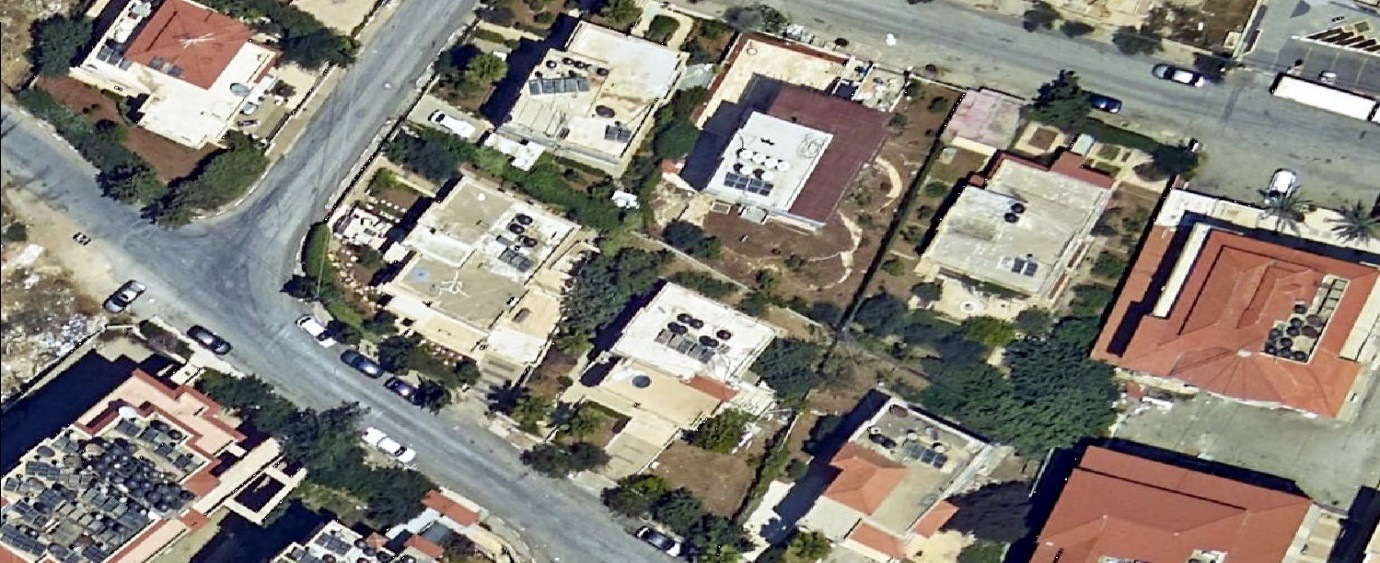 |
| --- |
| 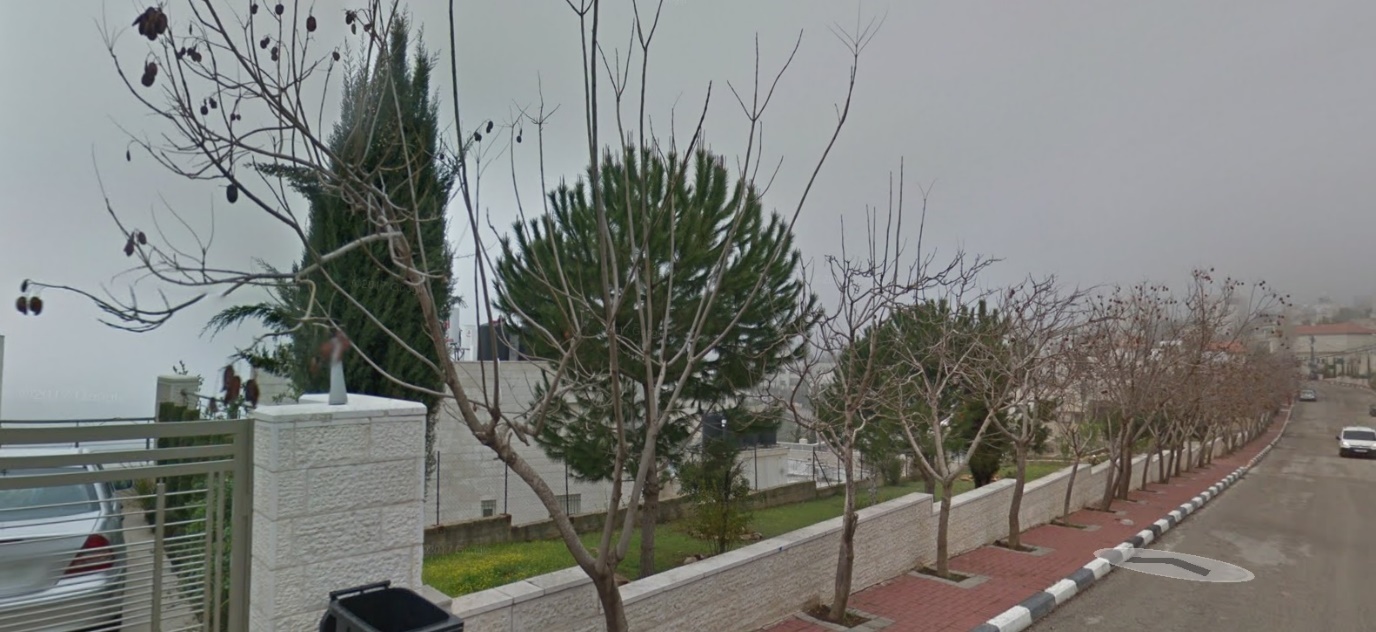 |
| Figure 10: Heterogeneous agricultural areas as appears in the orthogonal and Google street view image |
