# Supplementary material for: Contextual influences on chronic illness: A multi-level analysis in the twin cities of Ramallah and Al Bireh in the occupied Palestinian Territory
Source: Health Place. 2021 Nov;72:102677. doi: 10.1016/j.healthplace.2021.102677 (PMC8633762; doi:10.1016/j.healthplace.2021.102677)
Supplement: Multimedia component 2 [file mmc2.docx]

^[[1]](#footnote-1)^Table 1: The baseline and the final model including all the variables. Reporting log-odds and standard errors.

|  |  |  |
| --- | --- | --- |
|  | **Model 3 (Baseline)**  Log-odds(std.error) | **Model 4 (Final)**  Log-odds(std.error) |
| **Enumeration area characteristics** |  |  |
| Locality: (ref: Ramallah) |  |  |
| Al Bireh |  | 0.03 (0.12) |
| Refugee camps |  | 0.34 (0.27) |
| Political class: (ref: A&B) |  |  |
| Area C |  | -0.27 (0.17) |
| Green Space |  |  |
| Mixed Trees |  | **-0.04 (0.01)**** |
| Open space |  | -0.00 (0.00) |
| Crop trees |  | 0.01 (0.00) |
| **Individual characteristics** |  |  |
| Log(age) | 3.41(0.08)** | 3.42 (0.08)** |
| Female | -0.22 (0.05)** | -0.22(0.04)** |
| Education (ref: university) |  |  |
| 3^rd^ level school | 0.25 (0.05)** | 0.25 (0.05)** |
| 2^nd^ level school | 0.49 (0.05)** | 0.48 (0.05)** |
| 1^st^ level school | 0.72 (0.07)** | 0.72 (0.06)** |
| No education | 0.76 (0.07)** | 0.76 (0.07)** |
| Labour force (ref. employed) |  |  |
| Unemployed | 0.31 (0.12)** | 0.31 (0.12)** |
| Studying | 0.69 (0.12)** | 0.69 (0.11)** |
| Housework | 0.30 (0.06)** | 0.30 (0.06)** |
| other | 0.64 (0.05)** | 0.64 (0.05)** |
| Marital status (ref. Single) |  |  |
| Married | -0.08(0.06)* | -0.08 (0.06) |
| Widow-Divorce | 0.18 (0.08)* | 0.18 (0.08)* |
| Refugee | 0.15 (0.04)** | 0.15 (0.04)** |
| Health insured | 0.51 (0.06)** | 0.51 (0.06)** |
| Log(years of residence) | 0.02 (0.02) | 0.02 (0.02) |
| **Household characteristics** |  |  |
| Private car | -0.13 (0.04)** | -0.13 (0.04)** |
| Assets scale | -0.08 (0.03)** | -0.07 (0.03)** |
|  |  |  |
|  |  |  |
|  |  |  |
|  |  |  |
|  |  |  |
|  |  |  |
|  |  |  |

1. ** P_value<0.01, * P_value<0.05 [↑](#footnote-ref-1)
